# Comprehensive Cross-Sectional Study of the Triglyceride Glucose Index, Organophosphate Pesticide Exposure, and Cardiovascular Diseases: A Machine Learning Integrated Approach

**DOI:** 10.3390/toxics13020118

**Published:** 2025-02-01

**Authors:** Xuehai Wang, Mengxin Tian, Zengxu Shen, Kai Tian, Yue Fei, Yulan Cheng, Jialing Ruan, Siyi Mo, Jingjing Dai, Weiyi Xia, Mengna Jiang, Xinyuan Zhao, Jinfeng Zhu, Jing Xiao

**Affiliations:** 1 Nantong Key Laboratory of Environmental Toxicology, Department of Occupational Medicine and Environmental Toxicology, School of Public Health, Nantong University, Nantong 226019, China; lankeqing3910@gmail.com (X.W.); 2417320012@stmail.ntu.edu.cn (M.T.); zhaoxinyuan@ntu.edu.cn (X.Z.); 2 Nantong Hospital to Nanjing University of Chinese Medicine, Nanjing 210023, China

**Keywords:** TyG index, machine learning, network toxicology analysis, organophosphorus pesticides

## Abstract

Using NHANES data from 2003 to 2008, 2011 to 2012, and 2015 to 2020, we examined the relationship between urinary organophosphate pesticide (OPP) metabolites and the triglyceride glucose (TyG) index. The TyG index evaluates insulin resistance, a crucial factor in metabolic diseases. Linear regression analyzed urinary metabolites in relation to the TyG index and OPPs. An RCS (restricted cubic spline) model explored the nonlinear relationship of a single OPP metabolite to TyG. A weighted quantile regression and quantile-based g-computation assessed the impact of combined OPP exposure on the TyG index. XGBoost, Random Forest, Support Vector Machines, logistic regression, and SHapley Additive exPlanations models investigated the impact of OPPs on the TyG index and cardiovascular disease. Network toxicology identified CVD targets associated with OPPs. This study included 4429 participants based on specific criteria. Linear regression analysis indicated that diethyl thiophosphate was positively correlated with the TyG index. The positive correlation between OPP metabolites and the TyG index at low to moderate concentrations was confirmed by WQS and QGC analyses. The machine learning results aligned with traditional statistical findings. Network toxicology identified PTGS3, PPARG, HSP40AA1, and CXCL8 as targets influenced by OPPs. OPP exposure influences IR and cardiometabolic health, highlighting the importance of public health prevention.

## 1. Introduction

Organophosphorus pesticides (OPPs) are commonly utilized in farming, mostly highly or moderately toxic (some low toxic), and used to control pests, increase yield, and improve food nutrition. OPPs represent 34% of worldwide pesticide usage, and around 50,000 tons of OPPs are used annually in the United States [1]. According to a report issued by the World Health Organization and the United Nations Environment Programme (UNEP), globally, 3 million individuals are poisoned due to exposure to OPPs and similar substances, and 200,000 people succumb to such poisoning, with the majority being in developing countries. Exposure to OPPs and their metabolites primarily occurs via contaminated water and food [2]. Once the concentration of OPPs within the body reaches a certain level concerning the nervous system, it may potentially trigger Alzheimer’s disease (AD) [3] and Parkinson’s disease, among others [4]. It may also induce diabetes and metabolic disorders in the metabolic system [5,6].

Additionally, it may also lead to cardiovascular diseases (CVD) [7], and in extreme cases, it may even initiate cancer (including brain cancer, breast cancer, prostate cancer, bladder cancer, and colon cancer) [8,9,10]. In addition, studies have shown that the exposure of mice to pesticides is associated with disorders such as endocrine disruption, insulin resistance (IR), non-alcoholic fatty liver disease, and CVD [11,12]. IR constitutes the core link of metabolic disorders and is closely associated with diseases such as obesity, diabetes, and hypertension [13]. However, there are still relatively few studies on the changes between exposure to OPPs and IR, as well as on the indirect reflection of their direct relationship with cardiovascular diseases and metabolic syndrome. Consequently, research that connects OPP exposure to human cardiovascular and metabolism-related diseases holds significant research value.

IR refers to a condition marked by a reduced sensitivity and reaction to insulin [14]. Research has indicated that IR and related diseases are intimately associated with the occurrence of cardiovascular diseases [15]. Additionally, IR is acknowledged as a contributing factor to disease and a sign of unfavorable outcomes in individuals with CVD. Most conventional insulin resistance metrics, including the Homeostatic Model Assessment (HOMA) [16], Quantitative Insulin Sensitivity Check Index (QUICKI) [17], and Matsuda Insulin Sensitivity Index (MATSUDA) [18], are based on fasting plasma insulin levels. This method of determination has various constraints in clinical practice: (1) the levels of insulin in the plasma vary depending on how long the fasting period lasts; (2) interference related to hemolysis is prevalent; and (3) it is not a reliable measure for individuals with chronic diabetes and diminished insulin production. The triglyceride glucose (TyG) index has been recently suggested as a novel alternative for assessing insulin resistance. Multiple research studies have shown that its effectiveness in assessing IR is comparable to or better than that of HOMA-IR [19,20]. Furthermore, it has been found that the TyG index is associated with CVD [21,22], along with unfavorable clinical results in individuals experiencing acute coronary syndrome (ACS), heart failure (HF), ischemic stroke, and similar conditions. In most current studies, the TyG index is mostly used as a baseline indicator, covariate, or independent variable to explore the relationship with the corresponding outcome events. However, in this study, this index is used as an outcome indicator to reflect the impact on the body after OPP exposure [23,24,25].

At present, numerous studies have been conducted on OPPs and their metabolites. Guo et al. examined the relationship between OPP exposure and diabetes risk among American adults. The research findings suggest a positive correlation between OPP exposure and diabetes, indicating a possible linear trend between diethyl phosphate (DEP) levels and adult diabetes prevalence, with Bayesian Kernel Machine Regression (BKMR) results also supporting a positive correlation between mixed OPP metabolites and diabetes [26]. F. Glover and others studied the effect of OPP exposure on male erectile dysfunction (ED). Research has shown that being exposed to 3,5,6-trichloro-2-pyridinol (TCPy) correlates with a higher likelihood of ED in men [27]. At the same time, there is a wealth of studies regarding the TyG index. Qin Zhang and colleagues investigated the impact of the TyG index on patients with cardiovascular disease along with diabetes or prediabetes. The study indicates that among Americans with CVD and diabetes or prediabetes, the initial TyG index displays a U-shaped relationship with overall CVD mortality, highlighting its significance in prediction [28]. Pei et al. explored the connection between the TyG index and the quality of sleep. The research reveals that TyG has a moderate correlation with HOMA-IR (Spearman r = 0.51) and that a higher TyG index is linked to an increased occurrence of sleep disorders [29]. However, when considering previous cross-sectional studies of the population, relatively few investigations have utilized the TyG index as an outcome indicator to reflect the body’s metabolism and cardiovascular health to a certain degree. Hence, this study aims to fill this void and explore the correlation between OPPs, a common exposure, and the TyG index.

To summarize, we analyzed data from the National Health and Nutrition Examination Survey (NHANES) covering the epidemic cycles from 2003 to 2008, 2011 to 2012, 2015 to 2016, and 2017to 2020 to investigate the connection between exposure to OPPs and the TyG index. In this research, a weighted logistic regression model alongside a restricted cubic spline (RCS)–nonlinear model was used to investigate the impact of a single OPP on the TyG index. Furthermore, a weighted quantile and regression (WQS) model along with the quantile-based g-computation (QGC) method were utilized to assess the impact of mixed OPPs on the TyG index. Additionally, our research utilizes machine learning modeling along with the SHapley Additive exPlanations (SHAP) technique to obtain a deeper insight into the relationship between exposure to OPPs and alterations in the TyG index, as well as the associated diseases behind the changes in the index. Finally, this study employed a network toxicology analysis to investigate potential mechanisms linking exposure to OPPs with diseases associated with the TyG index.

## 2. Materials and Methods

### 2.1. Participant Group

NHANES is a continuous cross-sectional study aimed at evaluating the nutritional and health status of the civilian population in the United States. Every two years, a new cycle of NHANES was conducted with about 10,000 participants, and sociodemographic characteristics, health questionnaire data, body measurement, and biological samples tests were collected. Every participant gave their written informed consent before the study began.

Initially, we included a total of 65,906 study participants from six publicly available survey cycles (2003–2008, 2011–2012, 2015–2020). Subsequently, we excluded (1) 29,440 participants with age <20 years, (2) 25,686 participants without available data for urine OPs and TyG index, and (3) 6351 participants with covariates missing. Ultimately, 4429 participants were kept for the final correlation analyses.

### 2.2. Evaluation of the TyG Index

The TyG index is a numerical assessment of insulin resistance, determined by merging fasting blood glucose concentrations with triglyceride levels. Blood samples were collected from participants at baseline to measure fasting blood glucose (FSG) and triglyceride levels. The TyG index is determined using the formula TyG index = Ln [fasting TG (mg/dL) × fasting glucose (mg/dL)/2].

### 2.3. Condition of the Exposure

Urinary biomarker data for OPPs are provided in the Organophosphate Insecticides—Dialkyl Phosphate Metabolites—Urine section of the NHANES database. Six common urinary OPPs were covered, specifically dimethyl phosphate (DMP), diethyl phosphate (DEP), dimethyl thiophosphate (DMTP), diethyl thiophosphate (DETP), diethyl dithiophosphate (DEDTP), and dimethyl dithiophosphate (DMDTP). The data from the six cycles were combined, and only metabolites of OPPs with a detection rate of not less than 50%, i.e., DMP, DEP, DMTP, and DETP, were retained for subsequent statistical analysis (Table S1). For measurements falling below the lower limit of detection (LOD), the LOD divided by the square root of 2 was utilized as a substitute. In this assessment, a correction was applied utilizing creatinine, which was determined by dividing the concentration of OPPs by the level of urinary creatinine [30]. In addition, the NHANES Laboratory/Medical Technician Procedures Manual (LPM) provides more detailed information on the collection, measurement, and processing instructions for participant urine samples.

### 2.4. Covariates

The chosen covariates comprised sociodemographic information such as age, gender, marital status, race/ethnicity, the household poverty-to-income ratio, and education level. The anthropometric measures included body mass index (BMI), while personal behavioral factors considered were smoking status. Additionally, the status of chronic diseases was taken into account, including hypertension, diabetes mellitus, and cardiovascular disease. Information on age and gender was obtained from NHANES demographic data, while data on BMI, smoking status, and chronic diseases were obtained from questionnaires. The final sample size for inclusion was 4429 items. Race/ethnicity was classified into categories such as Mexican American, Other Hispanic, Non-Hispanic White, Non-Hispanic Black, and Other. Gender was divided into two groups: male and female. The educational attainment was classified into three groups: high school, below high school, and above high school. Household poverty income ratios (PIRs) were divided into three categories: low income (≤1.3), moderate income (1.3–3.5), and high income (≥3.5). Smoking status was transformed into categorical variables, i.e., smokers vs. non-smokers. Hypertension, diabetes, and cardiovascular disease were identified by self-reported medical history. For example, individuals who were told that they had hypertension were identified as hypertensive; individuals who self-reported diabetes were identified as diabetic. Body mass index (BMI) and age were treated as continuous variables.

### 2.5. Data Analysis

Continuous variables are expressed as means ± standard deviation, and the comparison of participant characteristics is conducted using Student’s *t*-test. Categorical covariates are shown as percentages and chi-square tests are used for comparisons. The creatinine-corrected urine OPP concentration data are processed by applying a logarithmic transformation to form a normal distribution. Pearson correlation coefficients are determined for OPP levels.

OPPs are categorized into quartiles, and three linear regression models are created to compute the β coefficient along with the associated 95% confidence intervals (CIs) to evaluate the relationship between OPPs and the TyG index. Model 1 does not account for any covariates. Model 2 expands upon Model 1 by additionally adjusting for the factors of age, gender, education level, marital status, race, and PIR value. Model 3 expands on Model 2 by also accounting for the impact of smoking, BMI, diabetes, hypertension, and cardiovascular disease. At the same time, a restricted cubic spline model featuring four knots is utilized to assess nonlinear relationships between each OPP and the TyG index.

To assess the overall impact of the four OPPs on the TyG index and the individual contribution of each OPP, a weighted quantile sum (WQS) regression analysis is employed to ensure that all selected OPPs in the model exhibit the same direction of effect on the TyG index. The WQS regression model assumes that the effects of all environmental exposures on outcomes are directionally consistent and linear and have a superimposed effect. In order to more comprehensively explore the relationship between OPPs and the TyG index, we have turned to a quantile-based g-calculation (QGC) approach. This method not only retains the straightforward logic of the WQS model but also integrates the adaptability of g-calculation, which can evaluate the cumulative impacts of various chemicals acting in diverse ways [31]. Statistical evaluation is conducted with R software (version 4.4.0). The “ggplot2” package, “gwqs” package, and “qgcomp” package are utilized to explore linear regression, WQS analysis, and QGC analysis, respectively. A significance level of *p* < 0.05 (two-sided) is established.

### 2.6. Machine Learning Analysis

Two previous population-level studies have demonstrated a correlation between organophosphate exposure and changes in the triglyceride glucose index with the development of CVD [32,33]. Consequently, the present study, which explores the correlation between organophosphate pesticide exposure and changes in the triglyceride glucose index, also sought to employ advanced machine learning models to enhance the comprehension of the effects of organophosphate pesticides on metabolism and cardiovascular disease. So, several algorithms are prominent when constructing machine learning models to understand the interaction among OPPs, TyG, and CVD-related health issues. XGBoost and Random Forest (RF), both ensemble methods, offer high accuracy and robustness by combining multiple decision trees. Support Vector Machines (SVMs) excel at classification tasks by finding optimal hyperplanes to separate data points. Finally, logistic regression (LR), a linear model, provides probabilistic predictions suitable for binary classification for CVD, CVD-related death, and the regression task of the TyG index. The choice of algorithm depends on the dataset distribution, with considerations for interpretability, computational cost, and desired prediction accuracy.

### 2.7. SHAP Interpretable Analysis

To enhance the interpretability of our machine learning models, we employ SHapley Additive exPlanations (SHAP). SHAP values provide insights into the contribution of each feature (OPPs, TyG index, age, BMI, etc.) to the model’s prediction for individual patients. This allows us to understand not only which factors are most influential in predicting CVD risk or the TyG index but also the direction and magnitude of their effects. For instance, SHAP can reveal whether higher OPP levels increase or decrease the likelihood of CVD and how this relationship varies across different patient subgroups. This interpretability is crucial for clinicians to trust and apply the model’s predictions in real-world healthcare settings, facilitating personalized risk assessment and treatment strategies.

### 2.8. Network Toxicology Analysis

The possible targets for OPPs were predicted with SwissTargetPrediction [34], and a search was performed in the GeneCards database [35] for protein targets linked to cardiovascular disease using the term “cardiovascular disease”. Venn diagrams were created using pertinent websites, and the overlapping genes derived from these diagrams were input into the STRING database [36] to build protein–protein interaction (PPI) models [37]. Afterward, the identified key intersections were evaluated using eight different topological metrics: Maximal clique centrality (MCC), Maximum neighborhood component (MNC), Degree, Edge percolated component (EPC), Closeness, Radiality, Betweenness, and Stress, by employing the analytics application known as cytoHubba within Cytoscape (Version 3.9.1). Simultaneously, analyses of the overlapping genes identified in Venn diagrams using the Kyoto Encyclopedia of Genes and Genomes (KEGG) and Gene Ontology (GO) were conducted utilizing the R software package (“org.Hs.eg.db”, “clusterProfiler”, and “DOSE”).

## 3. Results

### 3.1. Description of Study Participants

The overall features of the study group are summarized in Table 1. The study included 4429 American adults, comprising 2183 (49.3%) men and 2246 (50.7%) women. Most of the participants were aged 60 or below (68.9%), non-Hispanic white (42.8%), with a PIR > 1.3 (70.2%), an average BMI of 28.22 kg/m^2^, married (60.4%), smokers (74.8%), and the majority (75.9%) had an educational level of high school or above. Moreover, when comparing the group with a higher TyG index to the one with a lower index, there were statistically significant differences noted in terms of gender, age, educational attainment, marital status, race, body mass index (BMI), hypertension, diabetes, and cardiovascular disease status (Table 1). The highest detected concentration in urine was 13.73 ng/cg for DEP, followed by 11.57 ng/cg for DMP, 9.97 ng/cg for DMTP, and finally the lowest was 2.76 ng/cg for DETP.

### 3.2. The Relationship Between Individual Urinary Metabolites of Organophosphate Pesticides and the Tyg Index

**Figure 1:**
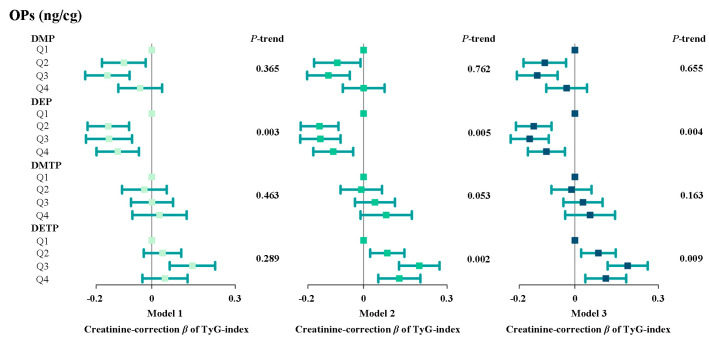
Relationship between urine OPP levels and TyG index for all participants. The square in the center of the line is the β value, and the width of the line represents the 95% CI. Model 1 did not include covariates; Model 2 included age, gender, education level, marital status, race, and PIR; Model 3 on 2 then includes smoking, BMI, diabetes, hypertension, and cardiovascular disease.

Figure 1 shows the results of the linear regression model adjusted for different covariates. This model evaluates the association between DMP, DEP, DMTP, DETP, and the TyG index. As shown in Model 1, in the initial model, compared with the low concentration (Q1), the TyG index of the medium and high concentration groups of the four OPP metabolites mostly shows an upward trend. As shown in Model 2, the DETP concentration is positively correlated with the TyG index. In comparison to Q1, the indices for groups Q2, Q3, and Q4 exhibit a rising trend, and this trend is statistically significant. In the comprehensive Model 3, DETP continues to show a significant positive association with the TyG index and higher DMTP levels are linked to an elevation in the TyG index.

### 3.3. Dose–Response Curve Between Opp Metabolites and Tyg Index

**Figure 2:**
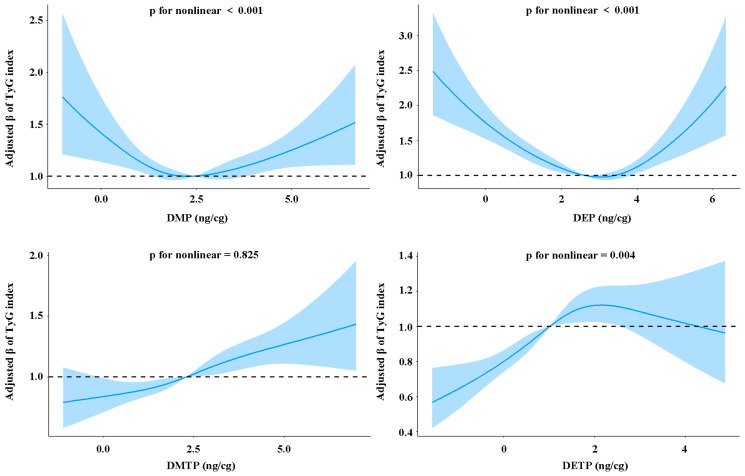
**Relationship between urinary OPP levels and TyG index based on RCS analysis.** Solid blue lines indicate ORs and blue shaded ranges indicate 95% CIs. Horizontal dashed lines represent reference ratios to 1.0.

Furthermore, we further evaluated the dose–response relationship between individual OPP metabolites and the TyG index (Figure 2). The RCS analysis and logistic regression yielded similar results. The RCS analysis shows that there is a significant nonlinear relationship between OPP concentrations (except DMTP) and the TyG index (*p* for nonlinearity < 0.05, Figure 2). For DETP and the TyG index, we observed a nonlinear dose–response curve characterized by an initial increase and then a gradually decreasing slope. In addition, DMP and DEP have an obvious U-shaped relationship with the TyG index. Generally speaking, in the medium and low concentration range, the concentration of OPP metabolites is significantly positively correlated with the TyG index.

### 3.4. The Relationship Between Mixtures of Urinary Organophosphate Pesticide Metabolites and the Tyg Index

**Figure 3:**
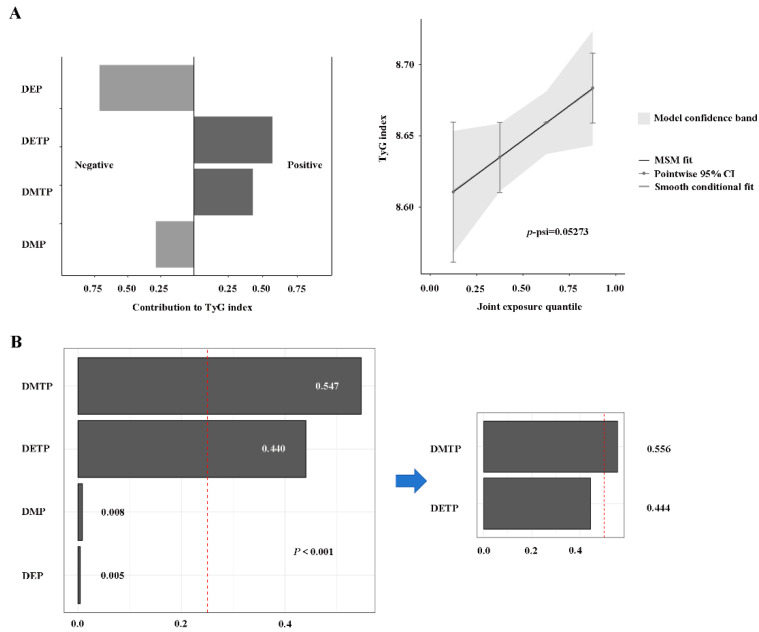
**The relationship between OPP combinations and TyG index in the QGC and WQS model.** (**A**) The result of the quantile-based g-computation. (**B**) The result of the weighted quantile and regression. All models were adjusted for demographic characteristics (age, gender, education level, marital status, race, PIR, and BMI), lifestyle (smoking), and disease status (diabetes, hypertension, and cardiovascular disease).

The interactive relationships among OPP metabolites were examined, and the Pearson correlation among these metabolites was analyzed, with the results presented in Figure S2. There are some interaction phenomena in DMP, DEP, DMTP, and DETP, indicating that the potential co-exposure patterns and multicollinearity among these OPP metabolites cannot be avoided in the conventional regression model. Consequently, we employ the WQS regression model and the QGC model to investigate the possible effects of co-exposure to OPPs, and the results are illustrated in Figure 3. In our examination, we modeled the upward trend of the WQS index and found a notable positive correlation between the WQS index and the TyG index (*p* < 0.001). In terms of the total effect of mixed exposure, the ranking weights of OPP metabolites show that DMTP (54.7%) and DETP (44.0%) are significant factors affecting the TyG index (Figure 3B). When the analysis is restricted to the negative direction, no significant association is observed in the overall population. In addition, the QGC analysis showed that an increase in the concentration of mixed OPP metabolites was associated with an increase in the TyG index (Figure 3A). Both DMTP and DETP show a positive correlation with the TyG index, which aligns with the findings of the WQS index.

### 3.5. Model Construction and SHAP Interpretable Result

**Figure 4:**
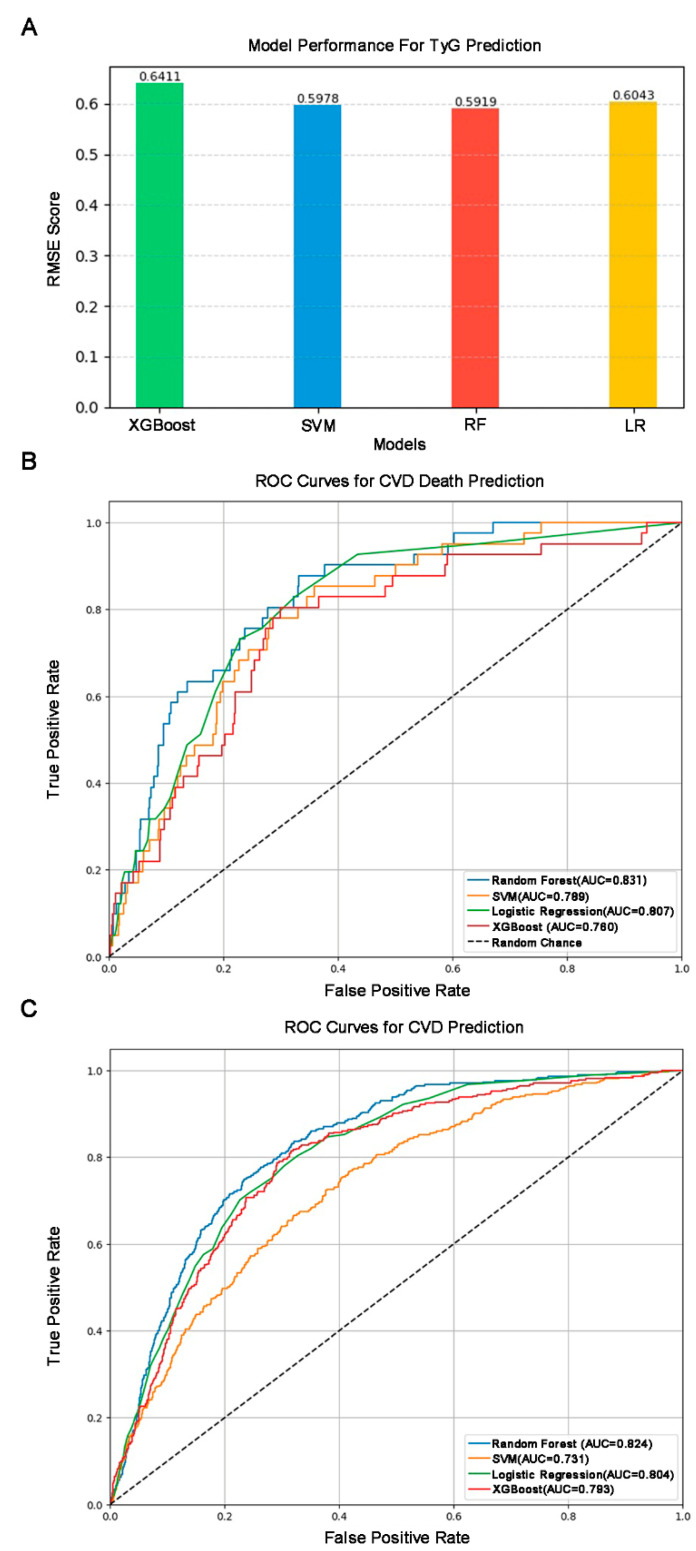
**Machine learning model performance and the ROC of the four machine learning models.** (**A**) Machine learning model performance for TyG index prediction. (**B**) ROC curves for CVD death prediction. (**C**) ROC curves for CVD prediction.

The regression task for the TyG index was performed with standard preprocessing data pipelines. The XGBoost was trained as the Root Mean Square Error (RMSE) with 0.641. The SVM obtains the RMSE with 0.597. The logistical regression has an RMSE of 0.604. The RF demonstrated the highest performance, showing an RMSE of 0.597 (Figure 4A). Regarding the prediction of CVD, XGBoost achieves an AUC (Area Under the Curve) of 0.793. The SVM records an AUC of 0.731. RF delivered the highest performance with an AUC of 0.824. The LR also demonstrates strong performance, with an AUC of 0.804 (Figure 4B). For the third prediction task, the CVD-related deaths among the four models had over 0.75 performance, while the RF achieved the best AUC performance at 0.831(Figure 4C). To enhance the clarity and model reliability, we recorded the specific model hyperparameter results (Table S2). The DeLong test for the pairwise comparison of different ROCs in Figure 4B,C was performed in fastDeLong. In Table S3, Random Forest (RF) attained the highest AUC (0.831) for predicting CVD mortality. Pairwise comparisons using DeLong’s test revealed that RF significantly outperformed SVM (*p* = 0.010) and XGB (*p* = 0.001), as indicated by the positive differences in the AUC (0.042 and 0.051, respectively) and 95% confidence intervals that did not include zero. Although RF showed a higher AUC than LR, the difference was not statistically significant (*p* = 0.123). Additionally, there were no significant differences among the remaining comparisons (SVM vs. LR, SVM vs. XGB, LR vs. XGB), indicating relatively similar performance among these models. As shown in Table S4, RF again achieved the highest AUC (0.824), with a significantly better performance than SVM (*p* = 0.0001) and XGB (*p* = 0.032). However, its advantage over LR was not significant (*p* = 0.186). Notably, SVM was significantly outperformed by LR (*p* = 0.0003) and XGB (*p* = 0.0016), reflecting a lower predictive performance for SVM on general CVD outcomes. The difference between LR and XGB was not statistically significant (*p* = 0.436). Overall, these results highlight the consistent strong performance of RF across both CVD mortality and broader CVD outcomes, with LR and XGB providing competitive AUC values in certain comparisons.

**Figure 5:**
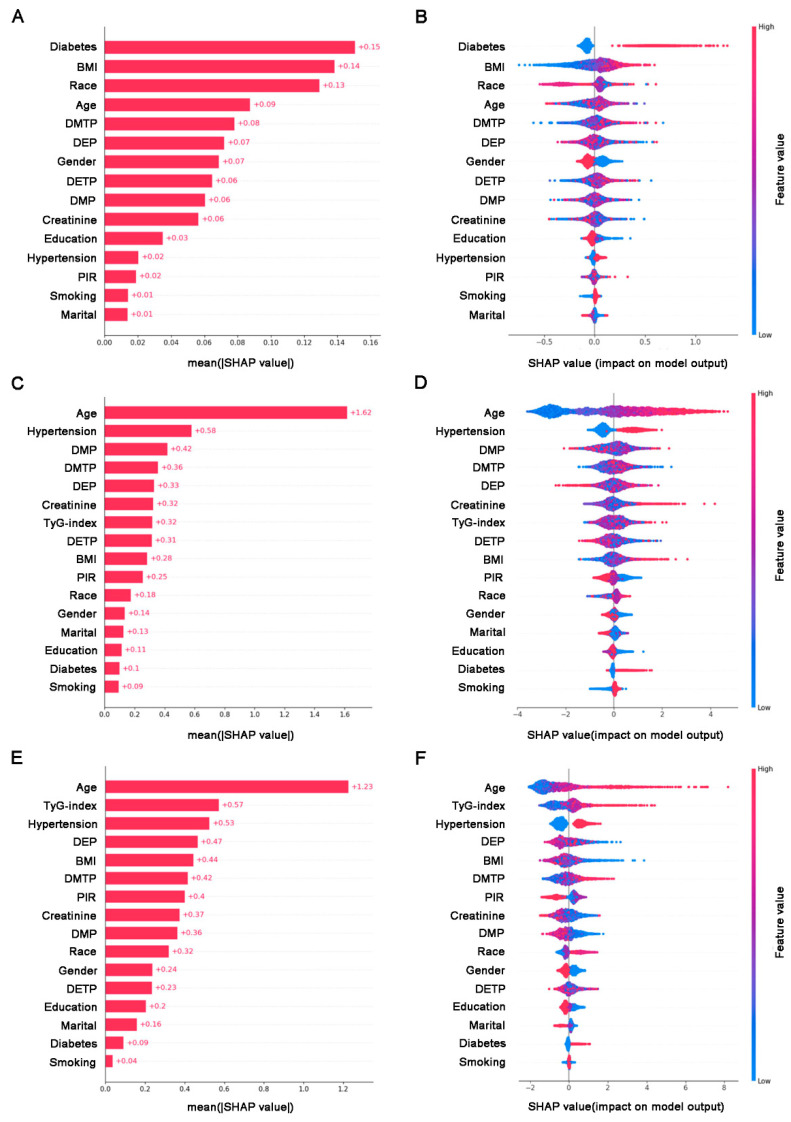
**The contribution of OPPs and baseline variables in predictive model.** (**A**) The SHAP features importance plot of OPPs and TyG index. (**B**) The SHAP summary plot of OPPs and TyG index. (**C**) The SHAP features importance plot of OPPs and CVD. (**D**) The SHAP summary plot of OPPs and CVD. (**E**) The SHAP features importance plot of OPPs and CVD-related deaths. (**F**) The SHAP summary plot of OPPs and CVD-related deaths.

To interpret the association among TyG, OPPs, and CVD-related health issues, we first deployed SHAP to reveal the correlation between OPPs and the TyG index. As matched by the statistical analysis, the OPPs have a positive relationship with the TyG index. In detail, DMTP has the highest ranking among all OPPs, contributing to higher exposure as the TyG index increases (Figure 5A,B). Furthermore, the game theory-based association between OPPs and CVD diseases with the background of significant factors are hypertension, BMI, and TyG index. The DMP, DMTP, and DEP have higher CVD rankings than the TyG index (Figure 5C,D). In the end, we explore the correlation between OPP exposure and CVD-related death based on the SHAP theory. DMTP has a positive correlation with CVD-related deaths. DEP and DMTP have a negative effect on CVD-related deaths (Figure 5E,F).

### 3.6. Details About Mechanisms and Targets

To gain a deeper insight into how OPPs, particularly DETP, affect cardiovascular-related diseases, we conducted functional enrichment analysis and built a PPI network at the convergence of OPP targets and proteins associated with cardiovascular diseases. We identified 59 target proteins by comparing the results from the Swisstargetprediction and Genecard databases (Figure S3A). The details of the KEGG and GO enrichment analysis results are presented in Figures S3B,C. Moreover, a protein–protein interaction (PPI) network featuring overlapping targets was established using the STRING database, as shown in Figure S3D. This network comprises 59 nodes and 150 edges, where each protein is illustrated as a node, and every edge symbolizes an interaction. The significance of overlapping targets within the interaction network was assessed using different algorithms available in Cytoscape software, such as MCC, MNC, Degree, EPC, Closeness, Radiality, Betweenness, and Stress. Utilizing the previously discussed algorithms, the overlapping targets were evaluated and prioritized, and the top five scoring proteins from each algorithm were recognized as potential targets for OPPs. Possible targets for OPPs might be further anticipated by intersecting candidate targets found through the eight algorithms, with key proteins identified through this approach, including PTGS3, PPARG, HSP90AA1, and CXCL8 (Figure S3E).

## 4. Discussion

This representative study suggests that exposure to OPPs is most likely associated with changes in the TyG index, implying a possible link between exposure to OPPs and the development of IR and additionally indicating a link between exposure to OPPs and the onset of cardiovascular or metabolic diseases. The key conclusion of this research is that contact with OPPs could be linked to alterations in the TyG index. The restricted triple spline showed a significant U-shaped relationship between DMP and DEP and the TyG index. Moreover, our results further validated that a notable positive relationship exists between co-exposure to OPPs and the TyG index, as assessed through the WQS model and QGC. This result was validated even after adjusting for other covariates. Meanwhile, Ashot Avagimyan et al. and Seyed Ali Nabipoorashrafi et al. systematically summarized the important role of the TyG index in the prevention of cardiology [38] and metabolic syndrome [39], which is seen to explore the relationship between exposure to OPPs and the TyG index, and thus indirectly predicting the relationship with the occurrence of metabolic diseases is highly feasible. To our knowledge, earlier research has not thoroughly investigated the connection between exposure to OPPs and the development of cardiovascular disease and metabolic syndrome, using the TyG index as a measure of outcomes. Consequently, this study may contribute valuable insights to this area of research.

The metabolic syndrome consists of six key elements, which are an accumulation of excess abdominal visceral fat, impaired IR or glucose tolerance, atherogenic dyslipidemia, increased blood pressure, and states that are pro-inflammatory and pro-thrombotic [40,41], which result in the manifestation of hyperlipidemia, diabetes mellitus, hypertension, and obesity, and increases the risk of cardiovascular disease [42]. It has been suggested that insulin resistance could be a causative factor in the onset of metabolic syndrome, which is marked by reduced tissue sensitivity or response to circulating insulin [43]. The non-biodegradable nature of OPPs and their multiple sources (including ingestion, inhalation, and dermal contact) contribute to the frequent exposure of humans to OPPs and, thus, adverse health effects. Exposure to OPPs has been shown to induce insulin resistance, which is a key factor in the development of cardiovascular and metabolic diseases [44]. Additionally, an increase in the TyG index is an effective indicator of the severity of insulin resistance [45]. Therefore, the TyG index can serve as an indirect measure of the connection between OPP exposure and the risk of cardiovascular and metabolic diseases. For instance, a study by Ou et al. found a positive correlation between exposure to OPPs and diabetes. Specifically, the adjusted odds ratio (aOR) for prediabetes among individuals with high urine concentrations of OPPs was 1.25 (95% confidence interval: 1.08, 1.46) compared to those with low urine concentrations [46]. Additionally, several studies have indicated that individuals with elevated levels of OPPs are at a higher risk of developing hypertension. This suggests a connection between exposure to OPPs and metabolic disorders [47]. Cardiovascular disease and metabolic syndrome are the two major diseases that threaten life and health in the United States. Therefore, our research employed the TyG index to investigate the connection between exposure to OPPs and the cardiovascular and metabolic health issues associated with insulin resistance, and our findings align with those of the aforementioned studies.

**Figure 6:**
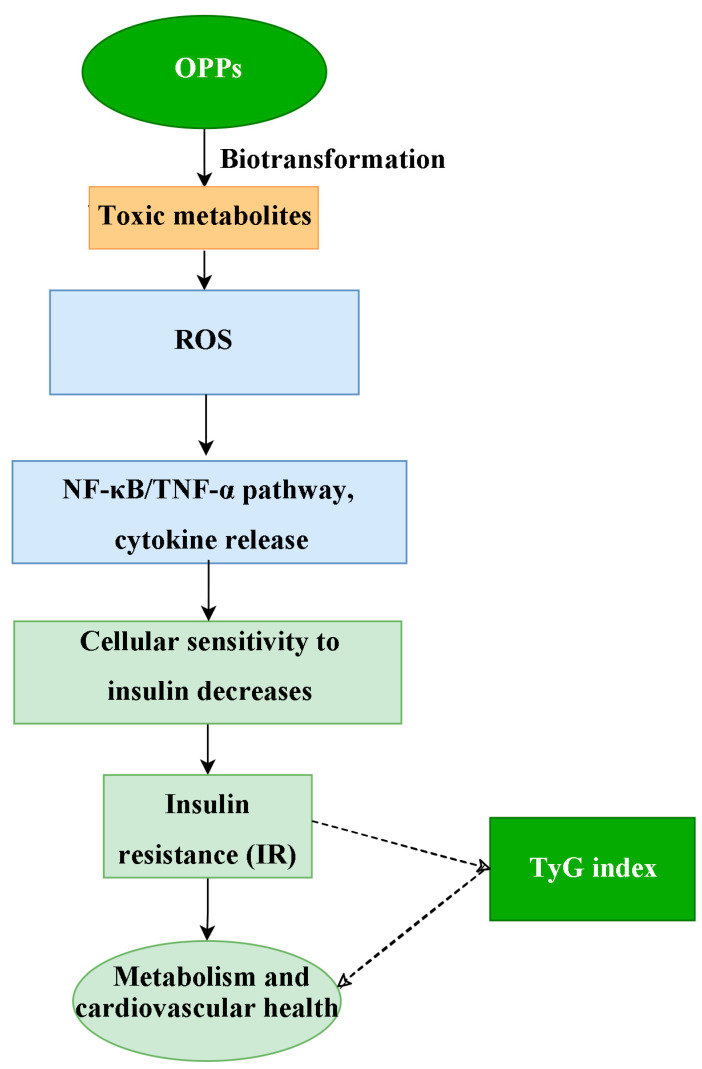
**Mechanistic pathway of OPP-induced insulin resistance.** OPPs, organophosphate pesticides; ROS, reactive oxygen species; NF-κB, Nuclear Factor-kappa B; TNF-α, Tumor Necrosis Factor-alpha; IR, insulin resistance; TyG index, triglyceride glucose index.

Notably, increased inflammatory/oxidative stress pathways are key mechanisms by which organophosphorus pesticide exposure induces metabolic disorders such as hypertension [48]. Oxidative stress and inflammatory responses induced by OPP may then promote vasoconstriction, increase vascular resistance, lead to vascular remodeling and stiffness, and consequently lead to endothelial dysfunction, which in turn affects metabolic homeostasis such as blood pressure regulation and ultimately leads to metabolic disorders such as hypertension [49]. Specifically, animal studies have shown that exposure to chlorpyrifos (CPF), one of the most widely used and dangerous organophosphorus pesticides, induces oxidative stress in vivo [50]. Similarly, chlorpyrifos exposure leads to elevated levels of reactive oxygen species (ROS), which can disrupt cellular antioxidant defenses in vitro [51,52]. In addition, exposure to the chlorpyrifos pesticide not only triggers oxidative stress but also enhances inflammatory responses by increasing the expression of pro-inflammatory cytokine genes (IL-1β and NLRP3) or by activating the NF-κB/TNF-α pathway in both in vivo and in vitro conditions [53,54]. Exposure to OPP leads to increased oxidative stress and inflammation in the body, as shown in Figure 6. OPP raises the levels of reactive oxygen species (ROS), which disrupts the cellular antioxidant defense mechanisms and activates inflammatory signaling pathways, such as the NF-κB/TNF-α pathway. This process also raises the expression of pro-inflammatory cytokines. The resulting oxidative stress and inflammation interfere with the insulin signaling pathway by blocking the phosphorylation of insulin receptor substrates. As a result, cellular sensitivity to insulin decreases, which can lead to insulin resistance [55,56]. In addition, the TyG index is a reliable indicator of insulin resistance, a key factor in the development of cardiovascular and metabolic diseases [45,57]. From another perspective, metabolic syndrome (MetS) manifests itself clinically in part as elevated levels of visceral fat, which enters the liver through the portal system and accumulates in the liver [58]. These interactions lead to higher levels of hepatic triglycerides (TG), reduced insulin reabsorption, and metabolic irregularities that eventually contribute to heightened IR [59]. Therefore, the TyG index can indirectly indicate the relationship between exposure to organophosphate pesticides (OPPs) and cardiovascular and metabolic diseases. Meanwhile, research has indicated that the TyG index is associated with direct indicators of insulin resistance. Studies have revealed that individuals with metabolic syndrome exhibit a notably higher average TyG index compared to those without the syndrome, and it also has correlations with various other health conditions [39,60]. For instance, research has demonstrated that a rise in the TyG index within a certain range elevates the likelihood of developing type 2 diabetes mellitus (T2DM) and cardiovascular disease (CVD) [61]; Ke et al. also revealed that an increase in the TyG index among the study participants heightened both all-cause mortality and CVD-related mortality, noting that a U-shaped TyG index particularly raised the risk of all-cause mortality, with a significant impact on CVD-related deaths [62]. These investigations once more offer robust evidence for the credibility of the findings of this research.

As previously stated, being exposed to OPPs is linked to the onset of metabolic disorders, with insulin resistance being a crucial factor in this process. There is compelling evidence for a significant correlation between insulin resistance, metabolic syndrome, and cardiovascular disease [63]. Therefore, it is feasible and plausible to use the TyG index, an assay of insulin resistance, to side-by-side explore the association of OPP exposure with the development of disorders such as metabolic syndrome and impaired regulation of lipid metabolism such as CVD. In addition, pertinent research has indicated correlations between the TyG index and arterial stiffness, as well as the likelihood of cardiovascular incidents [64,65]. Simal-Mendía et al. found that a higher TyG index correlated with decreased levels of high-density lipoprotein (HDL) and elevated triglycerides (TG) [66]. Various research findings indicate that the TyG index serves as a marker for individuals who are at risk of type 2 diabetes mellitus (T2DM) and prediabetes [67,68]. The diseases mentioned are primarily cardiovascular and metabolic, highlighting a clear correlation between the TyG index and these conditions.

The TyG index is widely recognized as being positively associated with the incidence of diabetes mellitus (DM) and hypertension within metabolic disorders [69], where insulin resistance (IR) is a key factor. Presently, the high insulin orthoglycemic clamp (HEC) technique is considered the gold standard for measuring insulin resistance, although it is both invasive and time-intensive [70]. As a straightforward alternative measure of insulin resistance, numerous studies have shown that the TyG index is effective in evaluating insulin resistance and preventing associated comorbidities. From a public health perspective, particularly for high-risk groups like agricultural workers, it is important to incorporate additional indicators related to insulin resistance and metabolic disorders, such as the TyG index, into regular medical checkups. We should also raise awareness about the dangers of exposure to OPPs to enhance public understanding of the TyG index and metabolic disorders. Furthermore, it is essential to strengthen wastewater treatment and soil remediation efforts, limit pesticide use, and improve environmental monitoring and the evaluation of relevant policies and regulations. In addition, the TyG index can be utilized not only to screen for potential metabolic abnormalities but also to monitor changes during the treatment of individuals with existing metabolic diseases. This information can assist in evaluating the effectiveness of treatment programs. Finally, clinicians can implement interventions based on changes in the TyG index to help prevent the development of complications. It is anticipated that research on the TyG index in public health and related fields will become more comprehensive, with an increased number of experiments devoted to exploring the underlying molecular mechanisms of OPP exposure, changes in the TyG index, and associated diseases.

The survey possesses multiple advantages. To begin with, the population data originated from a significant adult cohort in the United States, which increases the applicability of the results. Furthermore, a blend of conventional environmental mixed exposure assessments, cutting-edge machine learning techniques, and network toxicological analysis approaches were utilized to thoroughly investigate the impact of OPP exposure on diseases related to cardiovascular health and metabolic disorders.

However, despite these advantages, some limitations should also be considered. Firstly, it is important to highlight that NHANES is an observational study, which limits the capacity to establish a causal link between exposure to OPPs and the TyG index. Accordingly, further prospective cohort studies are required. Secondly, while SHAP values can account for interactions between features to some degree, they may overlook crucial interaction information, especially in cases involving complex relationships between variables. The targets identified through network toxicology should undergo experimental validation to provide stronger evidence. Furthermore, although this study assessed the potential effects of some confounders, such as demographics, disease status, and behavioral factors, there remain unmeasured confounders that could influence the observed associations. Ultimately, this study did not concentrate on a particular disease but rather on an index that, through the changes in the index, relatively comprehensively reflects the cardiovascular and metabolic health of individuals. However, the index does not fully represent individual metabolic and cardiovascular health. It is anticipated that the association observed between exposure to OPPs and the TyG index in this study will be confirmed through experimental research. Furthermore, conducting prospective cohort studies is essential to better understand the risks associated with OPPs and to develop more effective preventive measures to mitigate or prevent these hazards.

## 5. Conclusions

To summarize, our research indicates that exposure to OPPs (particularly DETP) is linked to an increased TyG index, an essential metabolic marker of insulin resistance that may indicate metabolic health to some degree and has a relationship with the development of cardiovascular diseases. This study is observational and still requires experimental validation. Consequently, the findings of this research demonstrate that exposure to OPPs could influence metabolism and cardiovascular health, investigate the underlying mechanisms, and identify potential target proteins, which hold significant importance for future empirical research and public health prevention strategies. Moreover, the TyG index has proven crucial in public health practices concerning cardiovascular and metabolic diseases linked to insulin resistance.

## Figures and Tables

**Table 1:** Demographic and clinical characteristics according to triglyceride glucose index level (*n* = 4429).

TyG Index Quartile	Total	Q1	Q2	Q3	Q4	*p*-Value
**Number**	4429	1106	1109	1107	1107	
**Gender (%) ^a^**						**<0.001**
Male	2183 (49.3)	441 (39.9)	541 (48.8)	579 (52.3)	622 (56.2)	
Female	2246 (50.7)	665 (60.1)	568 (51.2)	528 (47.7)	485 (43.8)	
**Age (years) group (%) ^a^**						**<0.001**
20–39	1480 (33.4)	541 (48.9)	382 (34.4)	307 (27.7)	250 (22.6)	
40–59	1571 (35.5)	354 (32.0)	374 (33.7)	400 (36.1)	443 (40.0)	
≥60	1378 (31.1)	211 (19.1)	353 (31.8)	400 (36.1)	414 (37.4)	
**Education level (%) ^a^**						**<0.001**
Below high school	1068 (24.1)	196 (17.7)	241 (21.7)	290 (26.2)	341 (30.8)	
High school	989 (22.3)	222 (20.1)	263 (23.7)	250 (22.6)	254 (22.9)	
Above high school	2372 (53.6)	688 (62.2)	605 (54.6)	567 (51.2)	512 (46.3)	
**Marital status (%) ^a^**						**<0.001**
Married/living with partner	2675 (60.4)	605 (54.7)	649 (58.5)	720 (65.0)	701 (63.3)	
Widowed/divorced/separated/	980 (22.1)	218 (19.7)	252 (22.7)	242 (21.9)	268 (24.2)	
Never married	774 (17.5)	283 (25.6)	208 (18.8)	145 (13.1)	138 (12.5)	
**Race (%) ^a^**						**<0.001**
Mexican American	682 (15.4)	104 (9.4)	165 (14.9)	186 (16.8)	227 (20.5)	
Other Hispanic	376 (8.5)	77 (7.0)	77 (6.9)	117 (10.6)	105 (9.5)	
Non-Hispanic White	1897 (42.8)	394 (35.6)	495 (44.6)	495 (44.7)	513 (46.3)	
Non-Hispanic Black	1008 (22.8)	410 (37.1)	262 (23.6)	193 (17.4)	143 (12.9)	
Other race	466 (10.5)	121 (10.9)	110 (9.9)	116 (10.5)	119 (10.7)	
**Household income (%) ^a^**						0.265
≤1.3 PIR	1321 (29.8)	309 (27.9)	335 (30.2)	318 (28.7)	359 (32.4)	
1.3–3.5 PIR	1681 (38.0)	417 (37.7)	425 (38.3)	428 (38.7)	411 (37.1)	
>3.5 PIR	1427 (32.2)	380 (34.4)	349 (31.5)	361 (32.6)	337 (30.4)	
**Body mass index (kg/m^2^),** **(median (25th, 75th)) ^b^**	28.22 (24.50, 32.70)	25.63 (22.41, 30.20)	27.79 (24.15, 32.02)	28.91 (25.53, 33.21)	30.00 (26.60, 34.19)	**<0.001**
**Smoking (%) ^a^**	3318 (74.8)	815 (73.7)	850 (76.6)	816 (73.7)	837 (75.6)	0.285
**Hypertension (%) ^a^**	1613 (36.4)	282 (25.5)	377 (34.0)	424 (38.3)	530 (47.9)	**<0.001**
**Diabetes (%) ^a^**	582 (13.1)	39 (3.5)	89 (8.0)	125 (11.3)	329 (29.7)	**<0.001**
**Cardiovascular diseases (%) ^a^**	502 (11.3)	81 (7.3)	132 (11.9)	125 (11.3)	164 (14.8)	**<0.001**
**Creatinine-adjusted urinary OPs** **(** **ng/cg** **), (median (25th, 75th)) ^b^**						
DMP	11.57 (3.80, 35.94)	11.41 (4.36, 30.05)	12.02 (3.93, 35.34)	11.49 (3.69, 37.05)	11.48 (3.55, 39.44)	**<0.001**
DEP	13.73 (3.49, 34.28)	15.27 (5.52, 34.87)	14.35 (3.48, 33.51)	12.80 (2.91, 33.93)	11.95 (2.80, 34.43)	**<0.001**
DMTP	9.97 (3.72, 30.22)	8.18 (3.38, 22.78)	9.59 (3.61, 30.07)	10.42 (3.85, 34.22)	12.30 (4.33, 35.24)	**<0.001**
DETP	2.76 (1.21, 6.34)	2.32 (1.04, 5.58)	2.69 (1.19, 6.21)	2.83 (1.24, 6.46)	3.22 (1.48, 6.84)	**<0.001**

^a^ Number of participants and percentage. The chi-square test was used for quartile groups of different TyG indexes. ^b^ Median (25th, 75th percentiles). The Kruskal–Wallis H test was used for quartile groups of different TyG indexes.

## Data Availability

The data used in this study can be downloaded for free in NHANES (https://wwwn.cdc.gov/nchs/nhanes/Default.aspx (accessed on 8 January 2025)). The code will be provided as required.

## Supplementary Materials

The following supporting information can be downloaded at: https://www.mdpi.com/article/10.3390/toxics13020118/s1, Table S1: detection rates of urine organophosphate 2003–2020; Table S2–1: comparison of XGBoost hyperparameters; Table S2–2: Random Forest hyperparameter comparison; Table S2–3: SVM (Support Vector Machine) hyperparameter comparison; Table S2–4: LR (logistic regression) hyperparameter comparison; Table S3: comparison of four CVD death prediction models; Table S4: four models’ AUC comparison in CVD prediction; Table S5: topological parameters associated with OPP targets for cardiovascular-related diseases; Figure S1: flowchart of the study population; Figure S2: Pearson correlation coefficients between urinary OPPs; Figure S3: the potential targets and mechanisms of OPPs were predicted through network toxicology analysis.
