# Supplementary material for: Comprehensive Cross-Sectional Study of the Triglyceride Glucose Index, Organophosphate Pesticide Exposure, and Cardiovascular Diseases: A Machine Learning Integrated Approach
Source: Toxics. 2025 Feb 1;13(2):118. doi: 10.3390/toxics13020118 (PMC11860532; doi:10.3390/toxics13020118)
Supplement: Supplementary file 1 [file toxics-13-00118-s001.zip › toxics-3433786-supplementary.pdf]

**This file includes:**

**Table S1.** Detection rates of Urine Organophosphate 2003-2020.

**Table S2-1.** Comparison of XGBoost Hyperparameters.

**Table S2-2.** Random Forest Hyperparameters Comparison.

**Table S2-3.** SVM (Support Vector Machine) Hyperparam Comparison.

**Table S2-4.** LR (Logistic Regression) Hyperparam Comparison.

**Table S3.** Comparison of Four CVD Death Prediction Models.

**Table S4.** Four Models' AUC Comparison in CVD Prediction.

**Table S5.** Topological parameters associated with OPPs targets for cardiovascular-related diseases.

**FigureS1.** Flowchart of the study population.

**FigureS2.** Pearson correlation coefficients between urinary OPPs.

**FigureS3.** The potential targets and mechanisms of OPPs were predicted through network toxicology analysis.

**Table S1.** Detection rates of Urine Organophosphate 2003-2020 (N = 4429).

| Urine Organophosphate (ng/ml) | 2003-2004 | | 2005-2006 | | 2007-2008 | |
|---|---|---|---|---|---|---|
| | <LLOD | Detection rate (%) | <LLOD | Detection rate (%) | <LLOD | Detection rate (%) |
| DMP | 287 | 50.17 | 410 | 35.33 | 490 | 35.10 |
| DEP | 264 | 54.17 | 451 | 28.86 | 526 | 30.33 |
| DMTP | 102 | 82.29 | 193 | 69.56 | 223 | 70.46 |
| DETP | 256 | 55.56 | 451 | 28.86 | 458 | 39.34 |
| DMDTP | 335 | 41.84 | 518 | 18.30 | 591 | 21.72 |
| DEDTP | 525 | 8.85 | 630 | 0.63 | 751 | 0.53 |
| | **2011-2012** | | **2015-2016** | | **2017-2020** | |
| | <LLOD | Detection rate (%) | <LLOD | Detection rate (%) | <LLOD | Detection rate (%) |
| DMP | 12 | 98.18 | 10 | 98.48 | 60 | 94.47 |
| DEP | 11 | 98.34 | 2 | 99.70 | 6 | 99.45 |
| DMTP | 32 | 95.16 | 65 | 90.09 | 142 | 86.91 |
| DETP | 185 | 72.01 | 256 | 60.98 | 505 | 53.46 |
| DMDTP | 294 | 55.52 | 343 | 47.71 | 616 | 43.23 |
| DEDTP | 630 | 4.69 | 634 | 3.35 | 1057 | 2.58 |

Note: LLOD: lower limit of detection.

**Table S2-1.** Comparison of XGBoost Hyperparameters.

| Hyperparameter | Range / Options | Model Setting | Description |
|---|---|---|---|
| n_estimators | 50 - 1000 | 175 | Number of boosting rounds (trees). |
| learning_rate | 0.01 - 0.3 | 0.1 | Shrinkage factor that scales newly added trees. |
| max_depth | 3 - 10 | 6 | Maximum depth of a tree. |
| min_child_weight | 1 - 10 | 1 | Minimum sum of instance weight (hessian) in a leaf. |
| gamma | 0 - 10 | 0 | Minimum loss reduction required to partition a leaf node. |
| subsample | 0.5 - 1.0 | 0.8 | Fraction of training data sampled per tree. |
| colsample_bytree | 0.5 - 1.0 | 0.8 | Fraction of features (columns) sampled per tree. |
| reg_alpha | 0 - 10 | 0 | L1 regularization term. |
| reg_lambda | 0 - 10 | 1 | L2 regularization term. |
| booster | gbtree, gblinear, dart | gbtree | Defines the base learner type for boosting |

Note: This table compares the same four models for general CVD Prediction. The AUC columns indicate model performance, while the difference in AUC, 95% CI, z - value, and p - value come from the DeLong test. The positive difference indicates the first model listed outperforms the second on AUC.

**Table S2-2.** Random Forest Hyperparameters Comparison.

| Hyperparameter | Range / Options | Model Setting | Description |
|---|---|---|---|
| n_estimators | 100 - 1000 | 420 | Number of trees in the forest. |
| max_depth | None - 50 | None | Maximum depth of each decision tree. |
| min_samples_split | 2 - 10 | 2 | Minimum number of samples required to split an internal node. |
| min_samples_leaf | 1 - 5 | 1 | Minimum number of samples required at a leaf node. |
| max_features | "auto", "sqrt", "log2", fraction | "auto" | Number of features to consider when looking for the best split. |
| bootstrap | True, False | True | Whether to use bootstrap samples. |

Note: This table compares the same four models for general CVD Prediction. The AUC columns indicate model performance, while the difference in AUC, 95% CI, z-value, and p-value come from the DeLong test. The positive difference indicates the first model listed outperforms the second on AUC.

**Table S2-3.** SVM (Support Vector Machine) Hyperparam Comparison.

| Hyperparameter | Range / Options | Model Setting | Description |
|---|---|---|---|
| C | 0.001 - 1000 (log scale) | 1.0 | Regularization parameter. |
| kernel | "linear", "poly", "rbf", "sigmoid" | "rbf" | Defines how data is mapped into a higher - dimensional space. |
| gamma | "auto", "scale", 10^-3 - 10^3 (log) | "scale" | Coefficient for RBF/poly/sigmoid kernels. "scale" = 1 / (n_features * X.var()) |
| degree | 2-5 (only if kernel="poly") | 3 | Degree of the polynomial kernel. |
| shrinking | True, False | True | speeds up convergence. |
| tol | 1e - 4 - 1e - 2 | 1e - 3 | Tolerance for the stopping criterion. |

Note: This table compares the same four models for general CVD Prediction. The AUC columns indicate model performance, while the difference in AUC, 95% CI, z - value, and p - value come from the DeLong test. The positive difference indicates the first model listed outperforms the second on AUC.

**Table S2-4.** LR (Logistic Regression) Hyperparam Comparison.

| Hyperparameter | Range / Options | Model Setting | Description |
|---|---|---|---|
| penalty | "l1", "l2", "elasticnet", "none" | "l2" | Type of regularization. |
| C | 0.001 - 1000 (log scale) | 1.0 | Inverse regularization strength. |
| solver | "liblinear", "lbfgs", "saga" | "lbfgs" | Optimization algorithm. "liblinear" suits small data or L1; "lbfgs" handles larger data and multiple classes. |
| max_iter | 50 - 1000 | 100 | Maximum number of iterations for convergence. |
| class_weight | None, "balanced" | None | Adjusts weights inversely proportional to class frequencies. |
| tol | 1e - 5 - 1e - 3 | 1e - 4 | Tolerance for stopping criterion. |

Note: This table compares the same four models for general CVD Prediction. The AUC columns indicate model performance, while the difference in AUC, 95% CI, z-value, and p-value come from the DeLong test. The positive difference indicates the first model listed outperforms the second on AUC.

**Table S3.** Comparison of Four CVD Death Prediction Models.

| Pair | AUC1 | AUC2 | Difference (AUC1 - AUC2) | 95% CI | z-value | p-value |
|---|---|---|---|---|---|---|
| RF vs. SVM | 0.831 | 0.789 | 0.042 | (0.012, 0.072) | 2.59 | 0.010 |
| RF vs. LR | 0.831 | 0.807 | 0.024 | (-0.006, 0.054) | 1.54 | 0.123 |
| RF vs. XGB | 0.831 | 0.780 | 0.051 | (0.022, 0.080) | 3.28 | 0.001 |
| SVM vs. LR | 0.789 | 0.807 | -0.018 | (-0.049, 0.013) | -1.18 | 0.241 |
| SVM vs. XGB | 0.789 | 0.780 | 0.009 | (-0.021, 0.039) | 0.59 | 0.556 |
| LR vs. XGB | 0.807 | 0.780 | 0.027 | (-0.003, 0.057) | 1.78 | 0.074 |

Note: This table compares four classification models for CVD Death Prediction. Each row shows a pair of models, their AUCs (AUC1 and AUC2), the difference in AUC, the 95% confidence interval (CI), and the DeLong test statistics (z-value and p-value). A positive difference indicates that the first model in the pair has a higher AUC.

**Table S4.** Four Models' AUC Comparison in CVD Prediction.

| Pair | AUC1 | AUC2 | Difference (AUC1 - AUC2) | 95% CI | z-value | p-value |
|---|---|---|---|---|---|---|
| RF vs. SVM | 0.824 | 0.731 | 0.093 | (0.060, 0.126) | 4.01 | 0.0001 |
| RF vs. LR | 0.824 | 0.804 | 0.020 | (-0.009, 0.049) | 1.32 | 0.186 |
| RF vs. XGB | 0.824 | 0.793 | 0.031 | (0.003, 0.059) | 2.14 | 0.032 |
| SVM vs. LR | 0.731 | 0.804 | -0.073 | (-0.106, -0.040) | -3.61 | 0.0003 |
| SVM vs. XGB | 0.731 | 0.793 | -0.062 | (-0.092, -0.032) | -3.16 | 0.0016 |
| LR vs. XGB | 0.804 | 0.793 | 0.011 | (-0.017, 0.039) | 0.78 | 0.436 |

Note: This table compares the same four models for general CVD Prediction. The AUC columns indicate model performance, while the difference in AUC, 95% CI, z - value, and p - value come from the DeLong test. The positive difference indicates the first model listed outperforms the second on AUC.Pair: The two models being compared; AUC1, AUC2: The respective AUC values of the models; Difference (AUC1 - AUC2): A positive difference means Model 1's AUC is higher; 95% CI: If this interval excludes 0, the difference may be statistically significant at $\alpha = 0.05$ (unadjusted); z-value: The standardized test statistic from DeLong's procedure; p-value: The probability of observing the measured difference.

**Table S5.** Topological parameters associated with OPPs targets for cardiovascular-related diseases.

| Gene names | MCC | MNC | Degree | EPC | Closeness | Radiality | Betweenness | Stress |
|---|---|---|---|---|---|---|---|---|
| PTGS2 | 1396 | 18 | 18 | 21.372 | 33.41667 | 6.02224 | 413.4608 | 1400 |
| PPARG | 1298 | 19 | 19 | 21.372 | 34.08333 | 6.05863 | 524.6733 | 1738 |
| CXCL8 | 1162 | 16 | 18 | 21.201 | 33.45 | 5.98585 | 596.0213 | 1520 |
| HSP90AA1 | 1118 | 15 | 17 | 20.891 | 32.0333 | 5.85849 | 476.0046 | 1750 |

Note: EPC, Edge Percolated Component; MCC, Mathews Coelation Coelicienjkt; MNC, Maximum Neighborhood Component

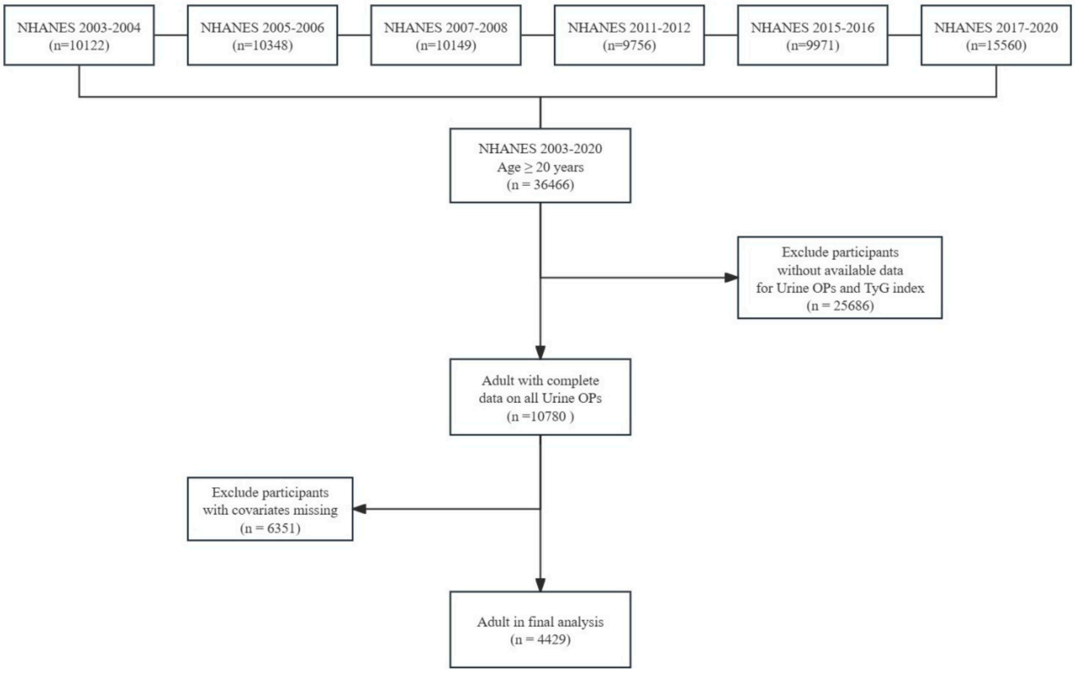


**FigureS1.** Flowchart of the study population.

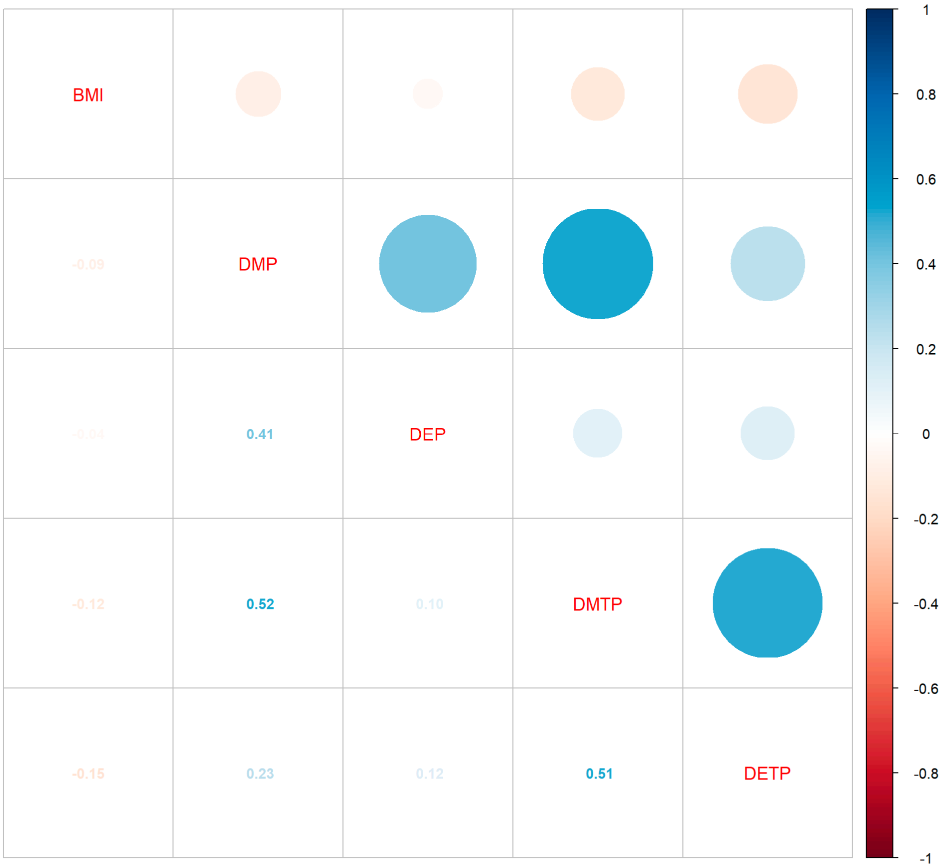


**FigureS2.** Pearson correlation coefficients between urinary OPPs.

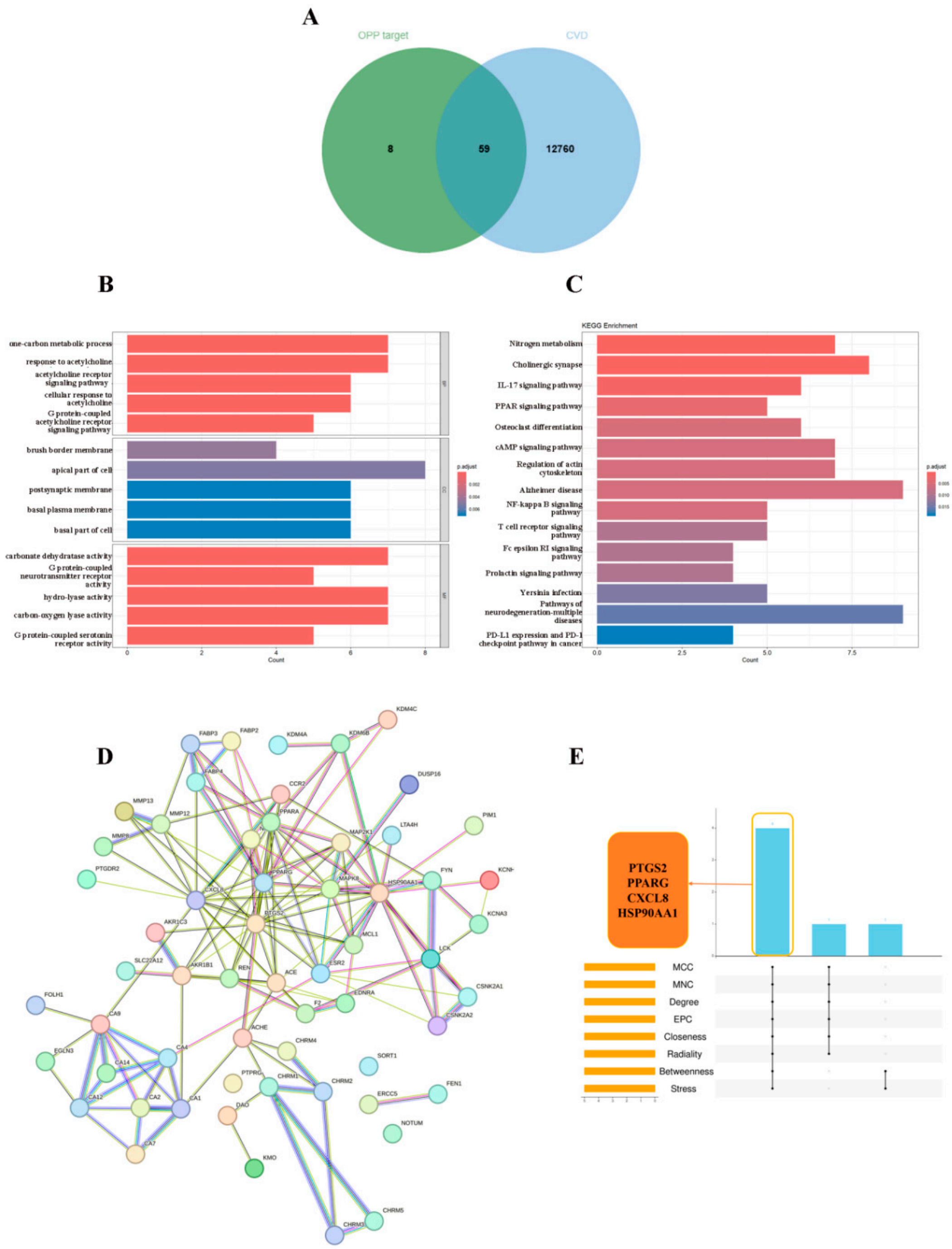


**FigureS3. The potential targets and mechanisms of OPPs were predicted through network toxicology analysis.** (A) Venn diagram illustrating the overlapping targets of OPPs and genes associated with cardiovascular-related diseases. (B) Bar plot of GO enrichment analysis. (C) Bar plot of KEGG enrichment analysis.(D)The PPI network of overlapping targets. (E) UpSet plot of 8 topological algorithms.
